# Supplementary material for: ANKS1B encoded AIDA-1 regulates social behaviors by controlling oligodendrocyte function
Source: Nat Commun. 2023 Dec 21;14:8499. doi: 10.1038/s41467-023-43438-1 (PMC10739966; doi:10.1038/s41467-023-43438-1)
Supplement: Supplementary file 1 — Supplementary Information [file 41467_2023_43438_MOESM1_ESM.pdf]

SUPPLEMENTARY FIGURES 1-10

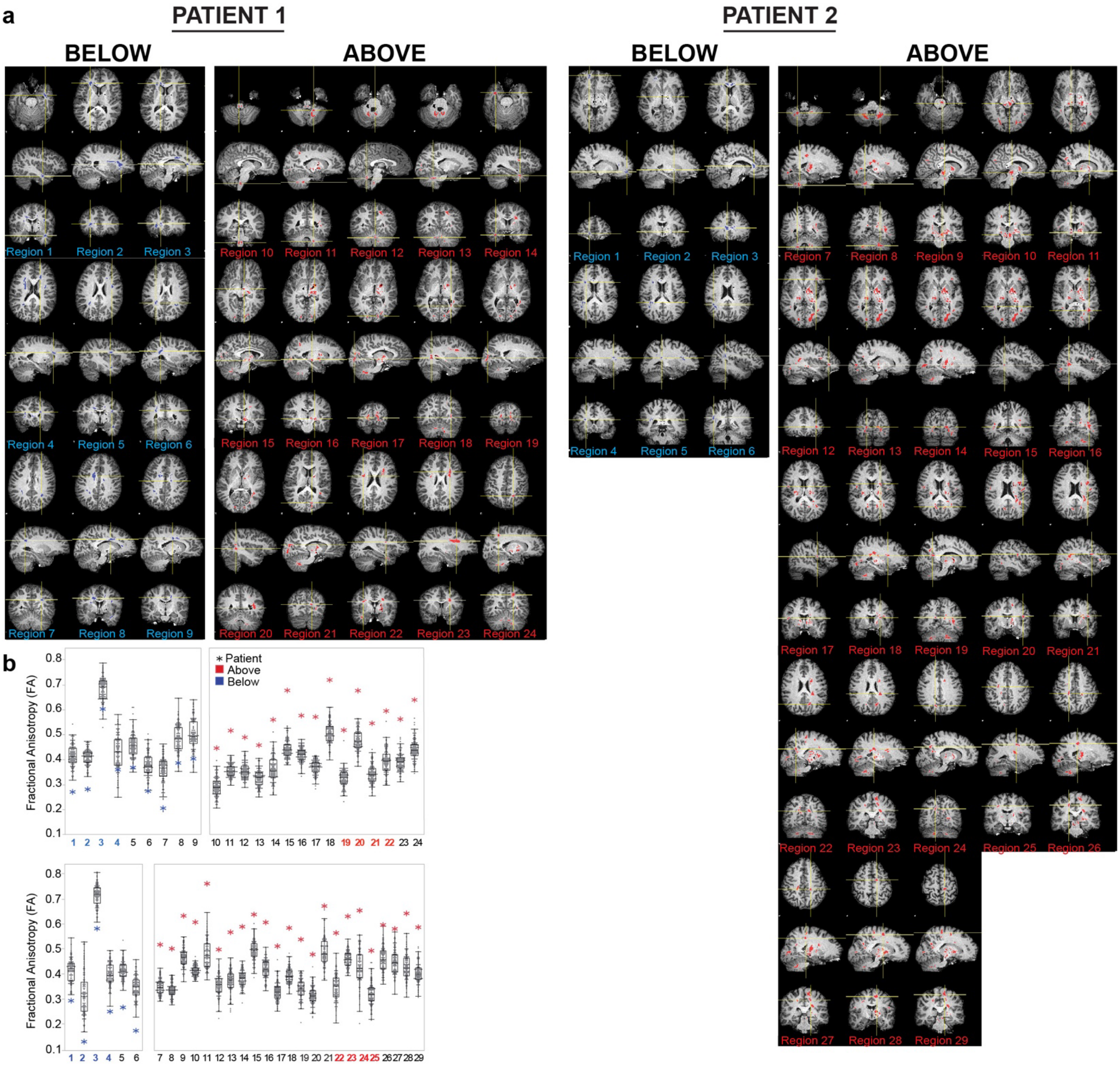

SUPPLEMENTARY FIGURE 1. Human DTI shows differences in ANDS patients compared to large control population. (a) MRI DTI images of all regions featured in Fig. 1d quantitation. **(b)** a duplicate of Fig. 1d.

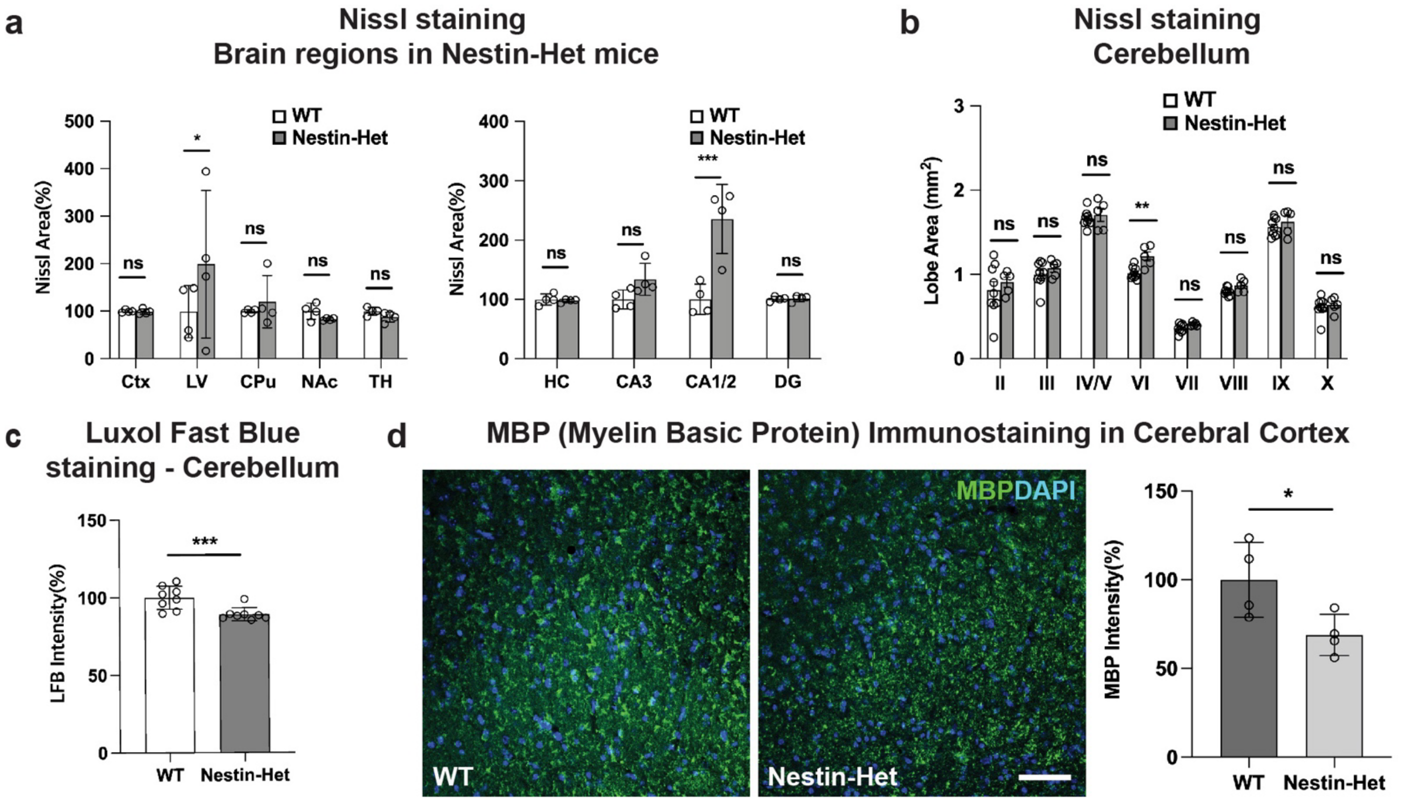

SUPPLEMENTARY FIGURE 2. Anatomical measures and MBP expression in an *Anks1b* haploinsufficiency mouse model. (a) Nissl staining of Nestin-Het (*n*=8) and WT (*n*=8) mice shows significant differences in (*left*) the lateral ventricle (LV) and (*right*) CA1/2 regions of the hippocampus. 18 sections were quantified from each mouse. (Ctx= cortex, CPu = caudate putamen, NAc = nucleus accumbens, TH = thalamus, HC = hippocampus, DG = dentate gyrus). **(b)** Nissl staining of cerebellar lobules in Nestin-Het (*n*=8) and WT (*n*=8) showing significant changes in the area (mm^2) of lobule VI. **(c)** LFB (Luxol fast blue) staining for myelin sheath components reveals decreased LFB density in cerebellum of Nestin-Het mice (*n*=9) compared to WT controls (*n*=10). **(d)** (*Left*) Immunostaining for MBP in cortex of Nestin-Het mice reveals (*right*) decreased myelination (Nestin-Het (*n*=8) and WT (*n*=8)). Scale bar = 20μm. (all panels) T-Tests; *, **, *** = *p* <0.05, <0.005, <0.0005.

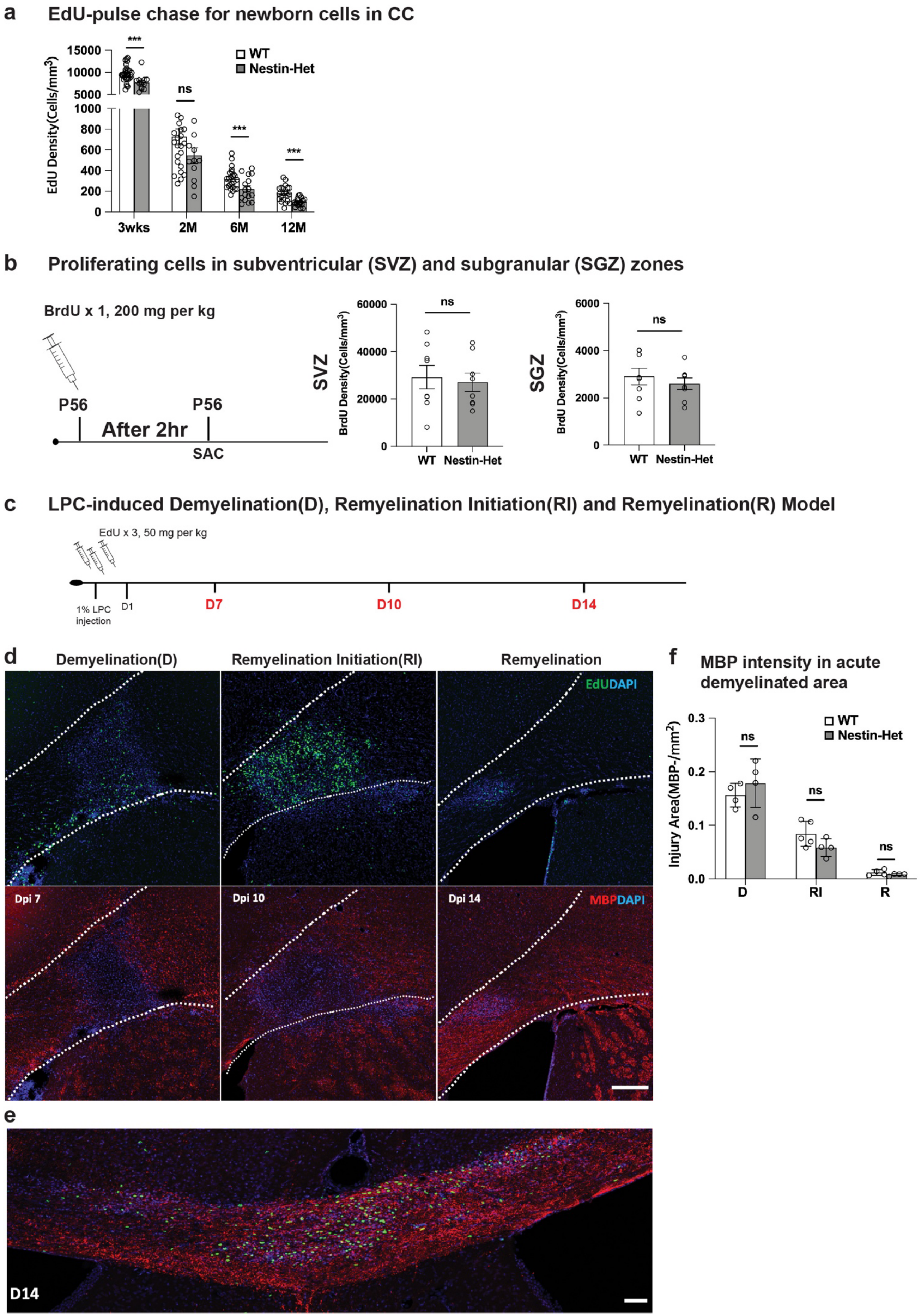
a
EdU-pulse chase for newborn cells in CC
EdU Density(Cells/mm3)
15000
10000
5000
1000
800
600
400
200
0
***
ns
***
***
WT
Nestin-Het
3wks
2M
6M
12M
b
Proliferating cells in subventricular (SVZ) and subgranular (SGZ) zones
BrdU x 1, 200 mg per kg
P56
P56
After 2hr
SAC
SVZ
BrdU Density(Cells/mm3)
60000
40000
20000
0
ns
WT
Nestin-Het
SGZ
6000
4000
2000
0
ns
c
LPC-induced Demyelination(D), Remyelination Initiation(RI) and Remyelination(R) Model
EdU x 3, 50 mg per kg
1% LPC injection
D1
D7
D10
D14
d
Demyelination(D)
Remyelination Initiation(RI)
Remyelination
EdUDAPI
Dpi 7
Dpi 10
Dpi 14
MBPDAPI
e
D14
f
MBP intensity in acute demyelinated area
Injury Area(MBP-/mm2)
0.3
0.2
0.1
0.0
ns
ns
ns
WT
Nestin-Het
D
RI
R

SUPPLEMENTARY FIGURE 3. No changes in neurogenesis or in MBP deposition following acute myelin lesions in *Anks1b*-deficient mice. (a) Related to **Figure 3(b, c)**, there were significantly fewer newborn cells overall in corpus callosum at 3 weeks, 6 months, and 12 months. Number of animals tested are same as in **Figure 3(b, c)**. **(b)** To measure neurogenesis, mice 6 months of age were injected with BrdU once into neurogenic zones. 2 hr after injections, mice were sacrificed, sectioned, and immunostained to identify newborn cells (BrdU+). Quantitation shows no significant differences in total number of newborn cells (Nestin-Het n=4 and WT n=4). **(c)** Protocol for experiment: 1% LPC (lysophosphatidylcholine) was injected into callosal regions to lesion myelin sheaths. EdU was injected IP to track newborn cells, and mice were sacrificed 7 (Demyelination-D), 10 (Remyelination Initiation-RI), and 14 days later (Remyelination-R). **(d)** Representative image showing (*upper*) newborn oligodendrocyte accumulation and (*lower*) MBP deposited in lesioned areas. Scale bar = 20μm **(e)** Newborn OPCs disperse throughout the corpus callosum by Dpi14. Scale bar = 100μm **(f)** Quantitation showing no changes overall in MBP deposited in lesioned areas. Number of animals tested, same as in **Figure 3(f-g)**. **(a, b, f).** Error bars reflect mean ± s.e.m. T-Tests; *, **, *** = $p < 0.05$, < 0.005, < 0.0005.

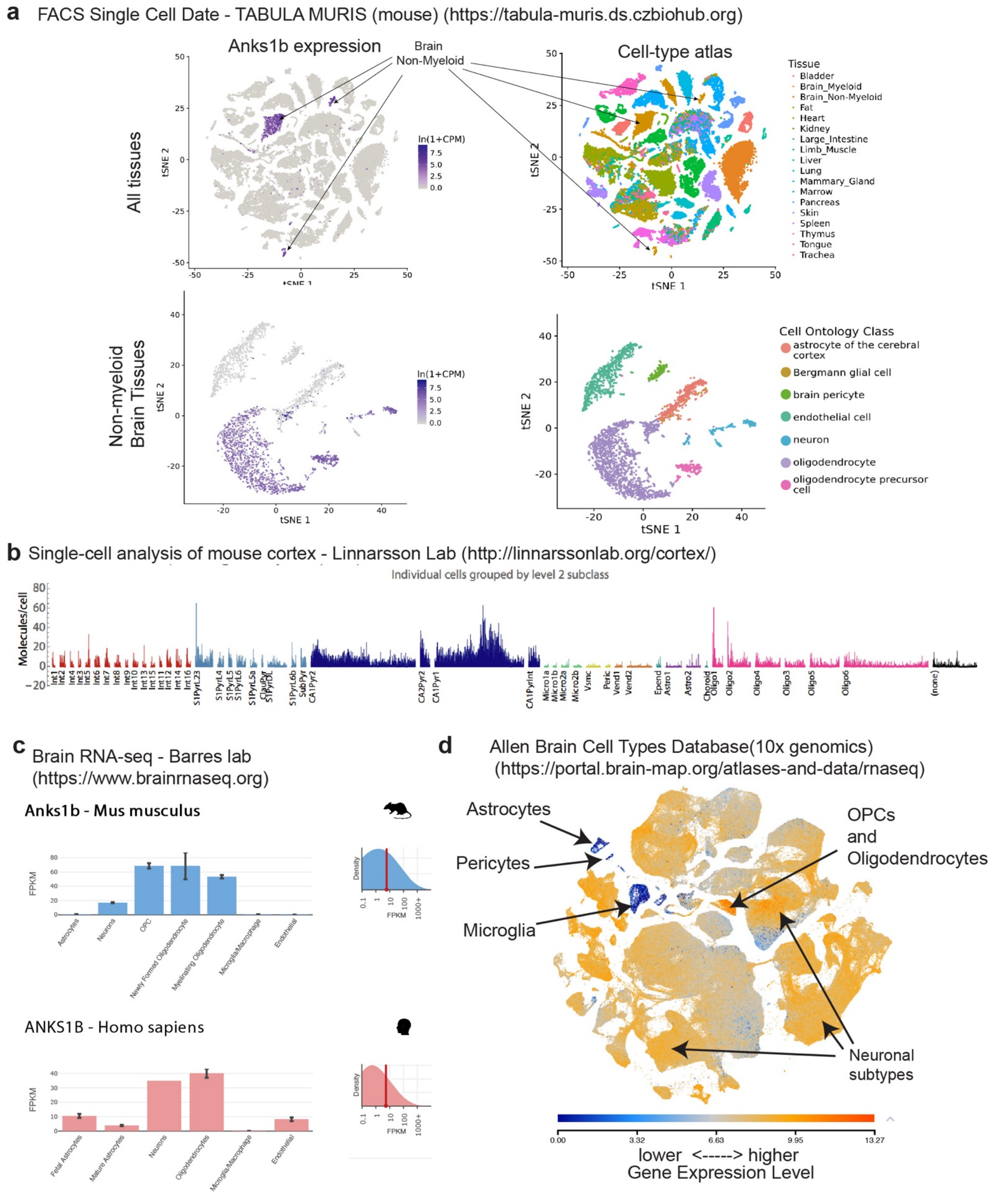

SUPPLEMENTARY FIGURE 4. AIDA-1 is expressed in oligodendrocyte lineage cells. Related to **Figure 4a.** Annotated tSNE and other single-cell transcriptomic expression charts taken from the indicated websites showing the expression of *Anks1b* in specific cell types.

a **Fluorescence *in situ* hybridization (*Anks1b*)- P14**

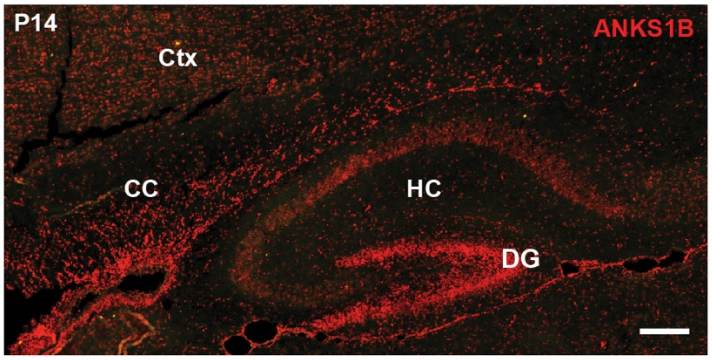

b ***Anks1b* expression in Oligodendrocytes (RT-PCR)**

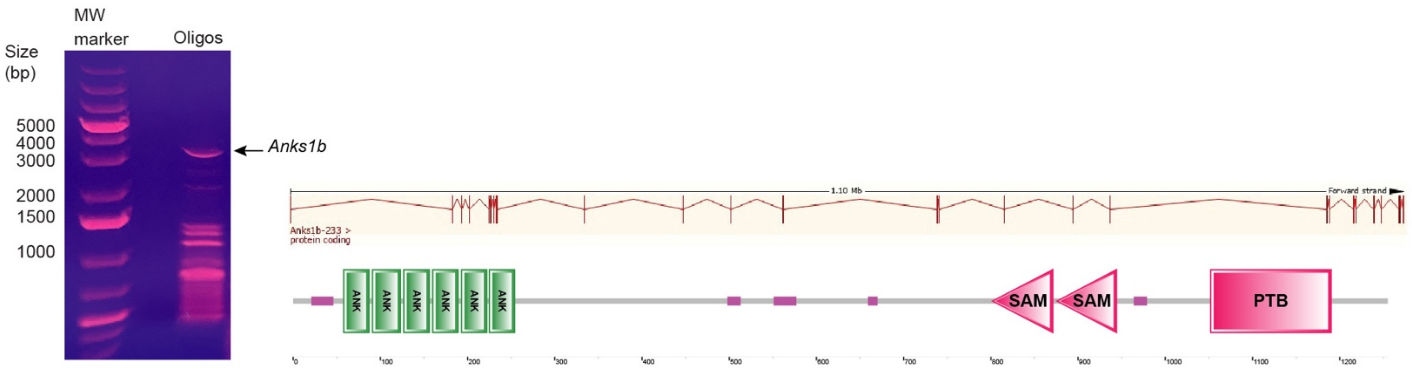

c **AIDA-1 developmental/region specific expression**

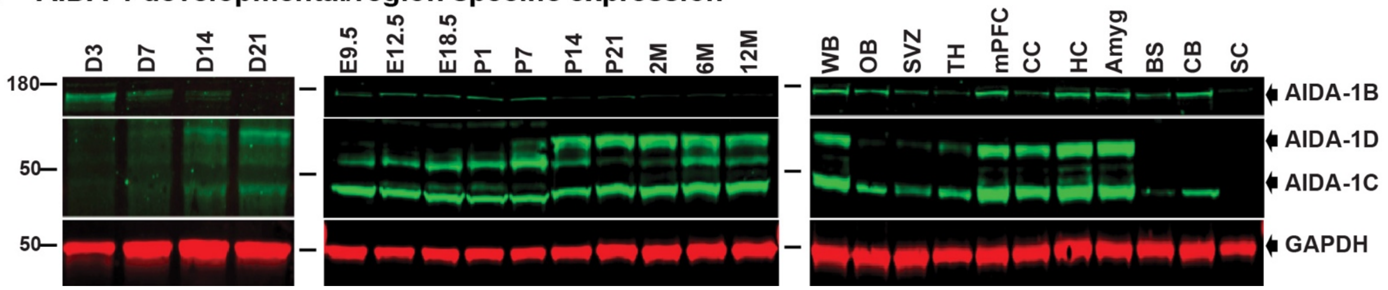

SUPPLEMENTARY FIGURE 5. AIDA-1 is expressed in myelin-rich regions and oligodendrocyte lineage cells. (a) *In situ* hybridization using fluorescently labelled probes targeting the 5' region of *Anks1b* reveals significant expression of larger (containing the Ankyrin repeats (see b.) transcripts in the cortex (Ctx), hippocampal (HC) CA1 and dentate gyrus, as well as in genu and splenium of corpus callosum (CC). Scale bar = 100μm **(b)** RT-PCR of *Anks1b* transcripts using gene-specific specific 3' primers and degenerate oligonucleotides for 5' priming using RNA extracted from enriched rat oligodendrocyte cultures. (*left*) DNA gel showing large *Anks1b* transcripts (AIDA-1B ~3kB) as well as smaller AIDA-1D transcripts. (*right*) sequencing of large 3kB transcript reveals a transcript encoding for large AIDA-1B containing 6 Ankyrin repeats. **(c)** Western blots using knockdown-verified AIDA-1 antibodies showing (*left*) AIDA-1 expression in rat primary cortical neurons at different days in culture, *(middle*) expression of AIDA-1 variants throughout development in total brain lysates, and (*right*) expression of AIDA-1 variants throughout brain regions. Representative of 3 Western blots.

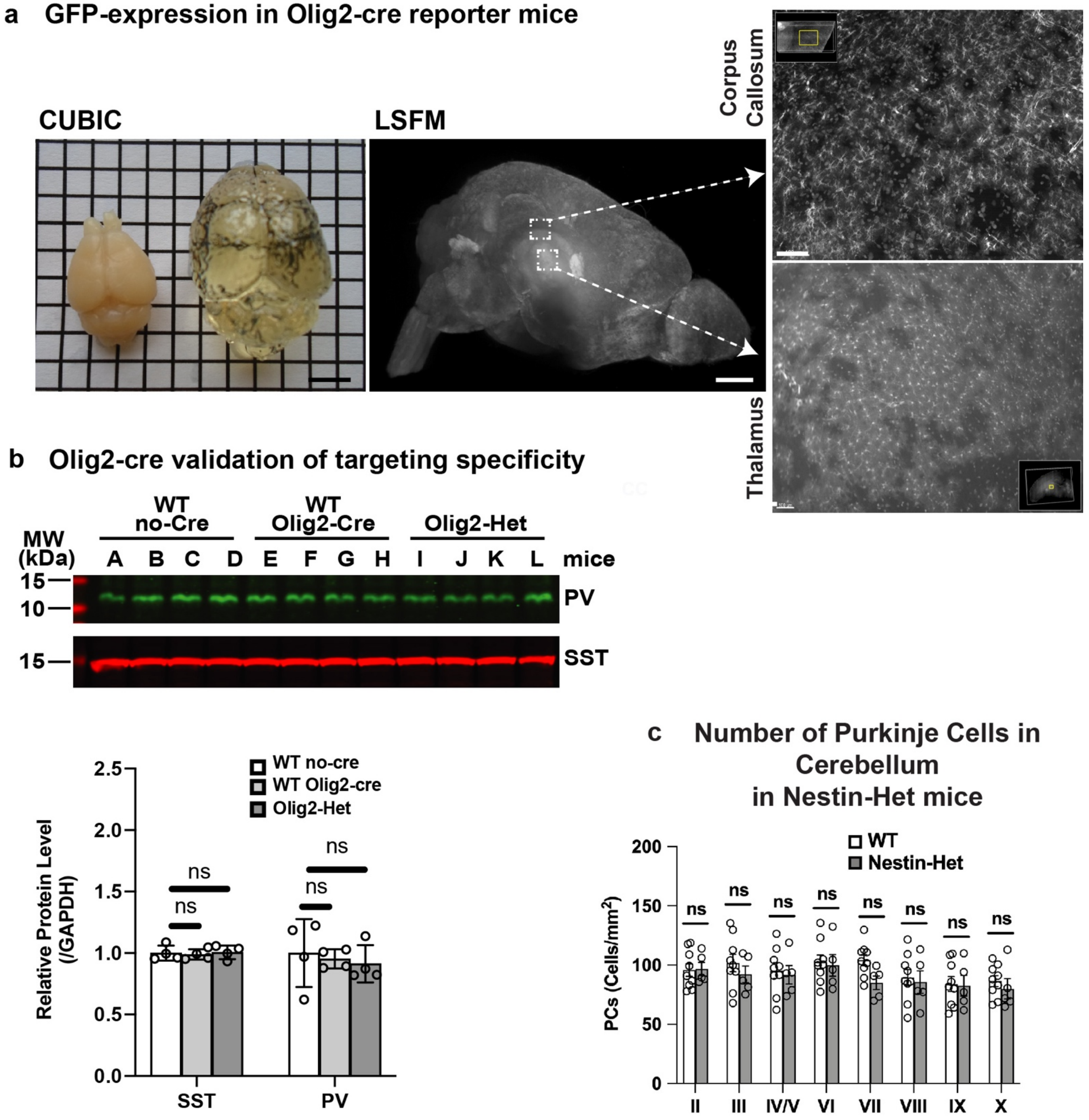

SUPPLEMENTARY FIGURE 6. Validation of specific targeting in *Olig2*-Cre mice. Olig2-reporter mice (*Olig2*-Cre X Rosa26-GFP) mice were generated, sacrificed, and dissected brains were subjected to CUBIC clarifying protocols. **(a)** Clarified brains were analyzed by light sheet microscopy (LSFM), which confirmed GFP expression in small cells with fine cloud-like processes throughout the brain, consistent with oligodendrocyte morphologies. Scale bar for whole brain image = 5mm, Scale bar for images on right = 100μm**. (b)** Olig2 expression was reported in a subset of interneurons early in development. However, no changes in PV (parvalbumin) or SST (somatostatin) expression were observed in Olig2-Het mice as assessed by Western blots, suggesting normal expression of interneurons. Each letter represents an independent mouse (*n*=4 different mice per genotype). Statistics and numbers same as **Figure 5f**. Error bars reflect mean ± s.e.m**. (c)** No changes were observed in the number of Purkinje cells (measured using calbindin staining) in Nestin-Het mice; T-tests.

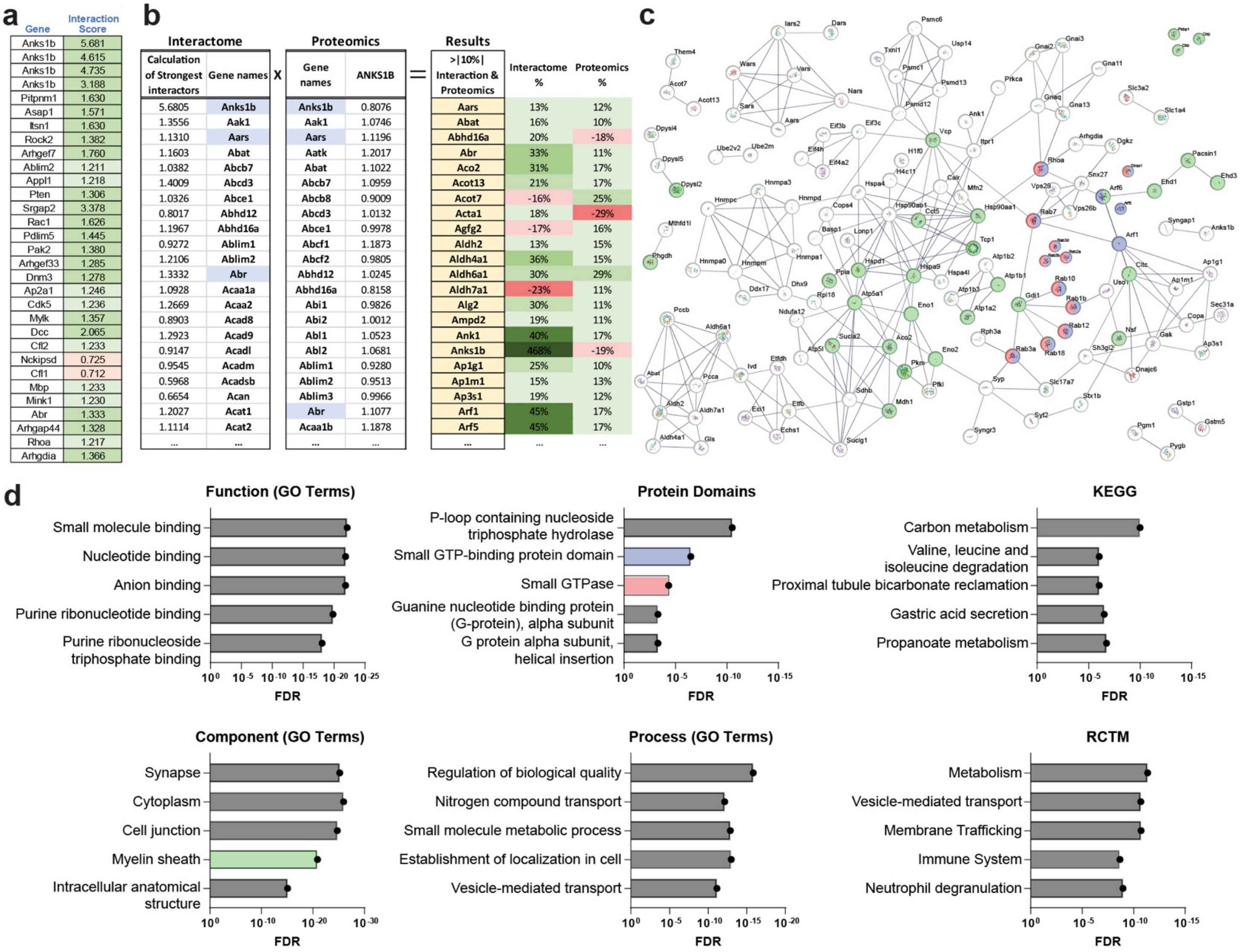

SUPPLEMENTARY FIGURE 7. Proteomic and Interactome analysis (a) Full list of select high scoring (>+20% light green, >+25% green, >-20% red) proteins from the interactome that are represented in the network model. **(b)** Extended diagram showing proteins that changed more than 10% compared to their respective controls for both the proteomics and interactome data. Full version in **Supp. Table 2**. **(c)** Unbiased Network model (StringDB) of the entire subset of proteins identified in the cross-analysis. Colors are consistent with results of the StringDB analysis output in part **(d),** which also shows extended results from the bioinformatic analyses. GO: gene ontology, FDR: false discovery rate

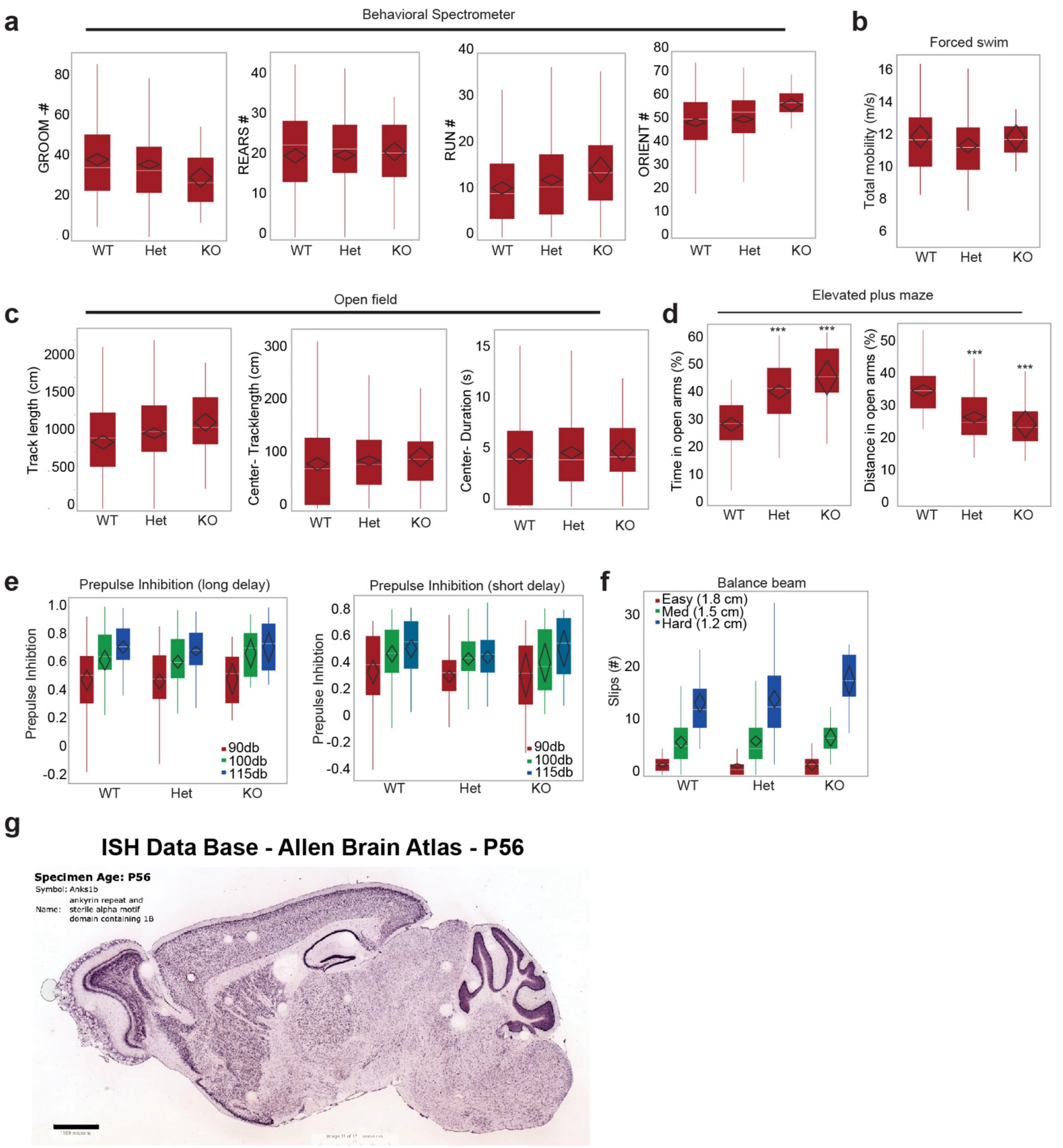

SUPPLEMENTARY FIGURE 8. Additional behaviors associated with *Anks1b Olig2*-Cre mice. a) Results from behavioral spectrometer. No differences in grooming, rearing, number of running events, or orienting were observed in *Anks1b* Olig2-Het or Olig2-KO mice compared to WT controls, suggesting normal risk assessment. **b)** No differences were observed in the Porsolt forced swim test of learned helplessness. **c)** No differences were observed in total track length, or total track length or time spent in center regions in open field testing. **d)** In the elevated plus maze, Olig2-Het and Olig2-KO mice spent (*left*) significantly more time in the open arms, but (*right*) covered significantly less track in open arms, revealing variable anxiety-related behaviors. **e)** No deficits in prepulse inhibition of the acoustic startle reflex were observed at any sound level at short or long intervals. **f)** Olig2-Het or Olig2-KO mice do not exhibit significant deficits in gross motor coordination as measured by slips on a balance beam. Box plots show 25th-75th quantiles (box), median (black

line), 95% confidence intervals (black diamond), and range (black whiskers). No significant sex differences were observed in any behaviors evaluated. For *Anks1b* Olig2-Het and Olig2-KO experiments, n= 53 WT, 82 Het, 12 KO. *** = $p < 0.0005$. **(g)** Micrograph of *Anks1b* expression (from Allen Brain Atlas) in sagittal slices of acute rat brains at p56, showing high expression in olfactory bulb, forebrain, hippocampus, and cerebellum. Scale bar = 1.2 mm.

a CaMKIIa-KO Mice- No Changes in Phenotype.

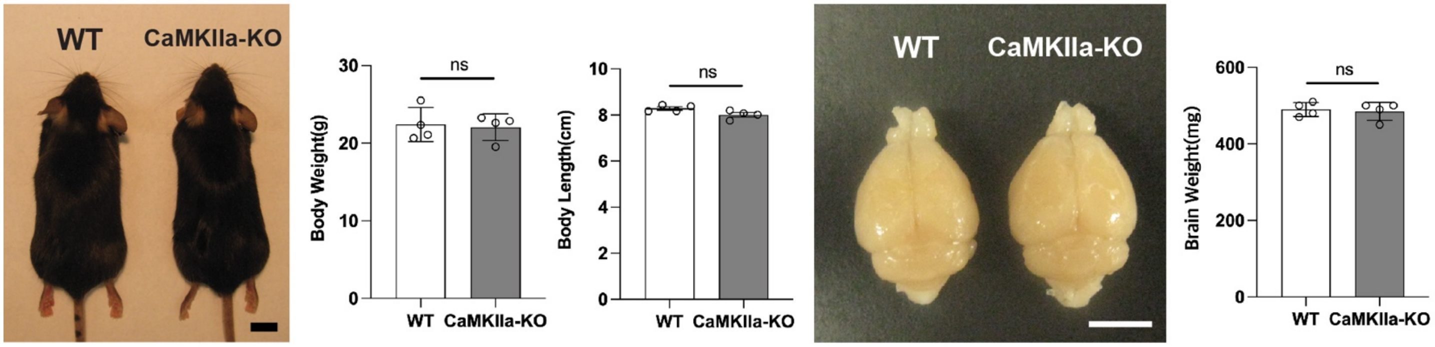

b CaMKIIa-KO Mice- No Changes in Brain Structure.

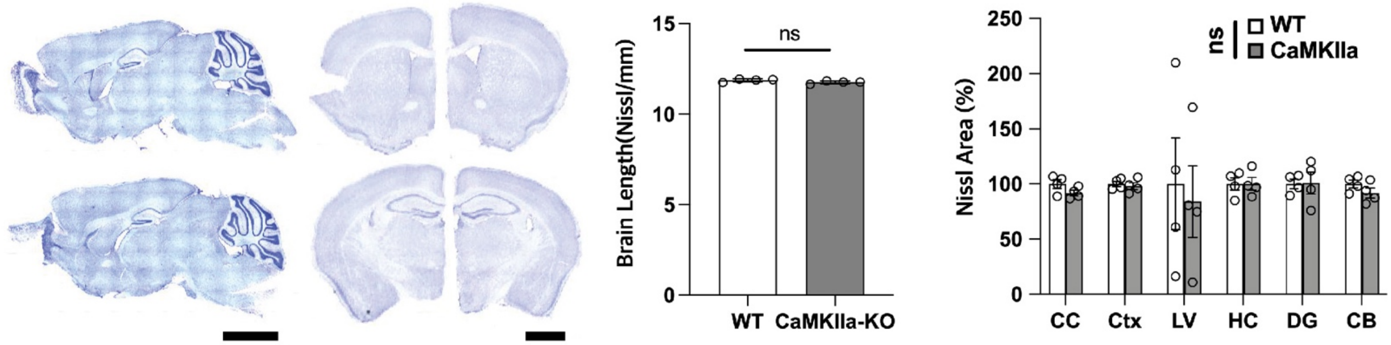

c CaMKIIa-KO Mice- No Changes in MBP expression.

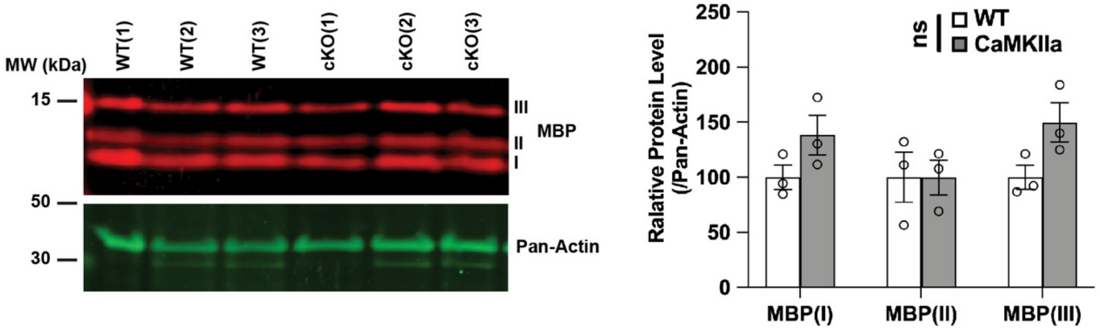

SUPPLEMENTARY FIGURE 9. CaMKIIa mice do not display the changes in gross anatomy or MBP expression present in Nestin-Het or Olig2-Het mice. (a) There were no changes in body measurements, scale bar = 10mm, or brain weight between CaMKIIa-KO or WT controls. Scale bar = 5mm. **(b)** Nissl staining of CaMKIIa-KO (*n*=8) and WT (*n*=8) mice shows no significant differences in different brain regions. Scale bar = 1mm.18 sections quantified from each mouse. CaMKIIa values normalized to WT. **(c)** Western blots of total brain lysates show that there were no changes in MBP expression (in any of the three quantified splice isoforms (I, II, or III) between genotypes. **(a-c)** Error bars reflect mean ± s.e.m; T-tests.

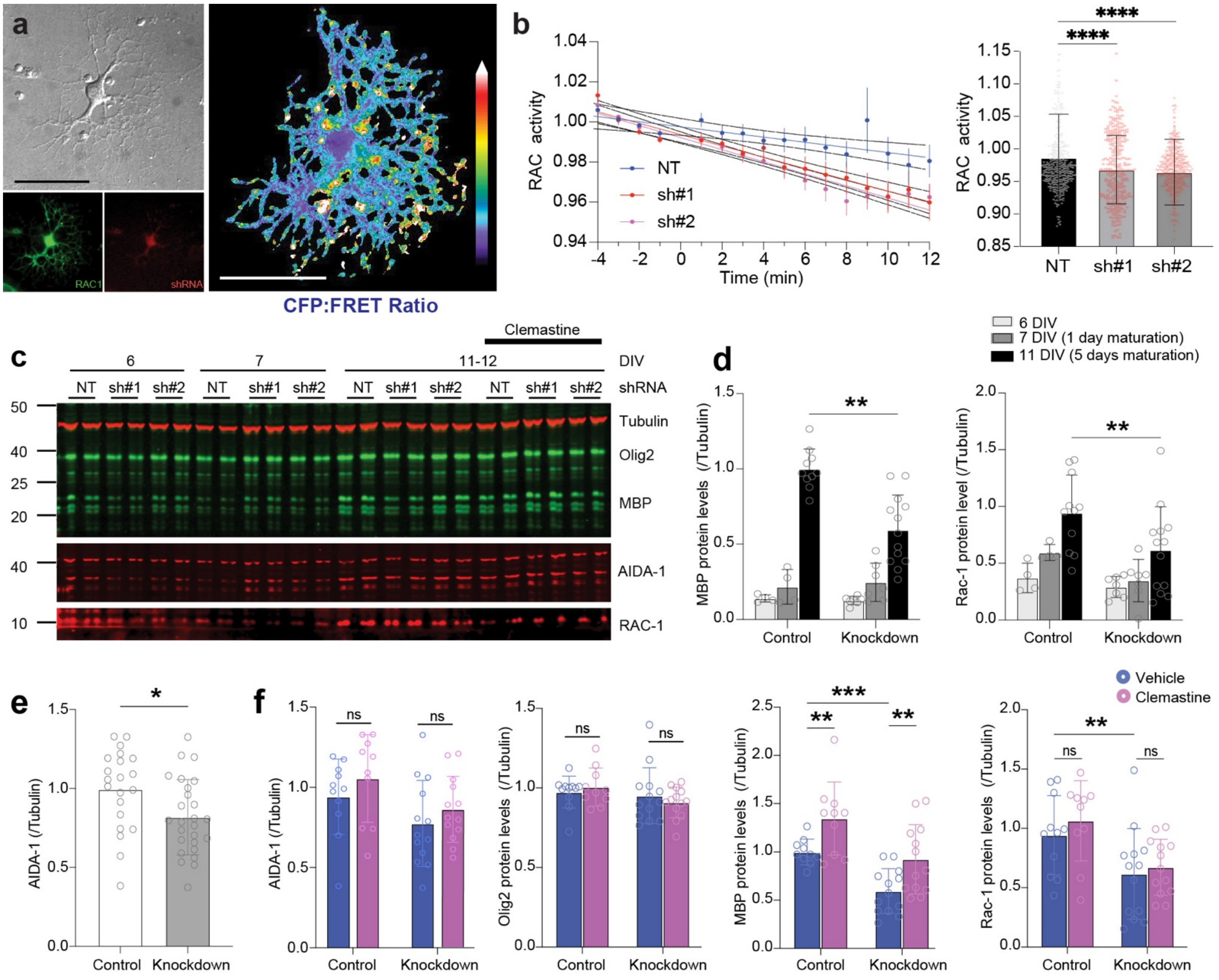

SUPPLEMENTARY FIGURE 10. Rac1 activation is significantly affected by AIDA-1 knockdown in rat oligodendrocytes. (a) FRET (Fluorescence Resonance Energy Transfer) imaging was completed in rat primary oligodendrocytes in the same way as for mouse cells in **Fig. 6a**. 2-3 days prior to transduction with FRET-based biosensors, rat OPCs were transduced with control (NT) or AIDA1-targeting shRNAs (sh#1, sh#2). Scale bar = 50 μm. **(b)** Plot presenting Rac1 activity over time. *n*=53 NT, 45 sh#1, and 64 sh#2-infected cells across 3 independent cultures; One-way ANOVA, with Dunnett's post hoc correction. **(c)** Representative western blot of primary cultures of rat OPCs (6, 7 DIV) and oligodendrocytes (11 DIV). **(d)** Transduction with shRNA for *Anks1b* decreases MBP expression in maturing oligodendrocytes. Results using sh#1 and sh#2 were not statistically different, so data for these two treatments was pooled (knockdown) for this and subsequent experiments. A reduction in Rac1 expression is also observed in mature *Anks1b*-deficient cells; One-way ANOVA with Dunnett's post hoc correction. **(e)** Targeting shRNAs significantly reduced AIDA-1 levels in oligodendrocytes; T-test. **(f)** Clemastine treatment increases the expression of MBP, but does not affect expression of AIDA1, Rac1, or the oligodendrocyte marker Olig2 (*n*=3 independent cultures); Two-way ANOVA with Tukey post hoc corrections. (**b, d, e, f**) Error bars reflect mean ± s.e.m. *, **, ***, **** = $p < 0.05$, <0.005, <0.0005, <0.0005.
