# Supplementary material for: ANKS1B encoded AIDA-1 regulates social behaviors by controlling oligodendrocyte function
Source: Nat Commun. 2023 Dec 21;14:8499. doi: 10.1038/s41467-023-43438-1 (PMC10739966; doi:10.1038/s41467-023-43438-1)
Supplement: Supplementary file 3 — Description of Additional Supplementary Files [file 41467_2023_43438_MOESM3_ESM.pdf]

Description of Additional Supplementary Files

File Name: Supplementary Data 1
Description: Spreadsheet containing full dataset of MRI and DTI analyses of Nestin-Het mouse brains. First tab contains the full dataset of the volumes (mm^3) of specific brain regions calculated from T2-weighted MRIs of ex vivo brain tissue from 10 female Anks1b Nestin-Het and 10 female WT controls. Second tab contains full dataset of the FA (fractional anisotropy) analyses of specific brain regions from in vivo diffusion tensor imaging (DTI) of 5 female Anks1b Nestin-Het and 5 female WT controls. Experimental groups and statistical analyses are detailed in each tab.

File Name: Supplementary Data 2
Description: Spreadsheet containing the full list of proteins identified in proteomic studies of Anks1b hippocampal synapses (Carbonell AU et al 2023), and Anks1b interactome studies (Carbonell AU et al 2019), along with full set of proteins that overlap both datasets.

File Name: Supplementary Data 3
Description: Full list of Antibodies, reagents, mouse lines, and genotyping primers used in this study.
