# Supplementary material for: ANKS1B encoded AIDA-1 regulates social behaviors by controlling oligodendrocyte function
Source: Nat Commun. 2023 Dec 21;14:8499. doi: 10.1038/s41467-023-43438-1 (PMC10739966; doi:10.1038/s41467-023-43438-1)
Supplement: Supplementary file 7 — Reporting Summary [file 41467_2023_43438_MOESM7_ESM.pdf]

nature portfolio

Corresponding author(s): Bryen A. Jordan
Last updated by author(s): 11/6/2023

Reporting Summary

Nature Portfolio wishes to improve the reproducibility of the work that we publish. This form provides structure for consistency and transparency in reporting. For further information on Nature Portfolio policies, see our Editorial Policies and the Editorial Policy Checklist.

Statistics

For all statistical analyses, confirm that the following items are present in the figure legend, table legend, main text, or Methods section.

n/a	Confirmed	
☐	☒	The exact sample size (*n*) for each experimental group/condition, given as a discrete number and unit of measurement
☐	☒	A statement on whether measurements were taken from distinct samples or whether the same sample was measured repeatedly
☐	☒	The statistical test(s) used AND whether they are one- or two-sided<br>*Only common tests should be described solely by name; describe more complex techniques in the Methods section.*
☒	☐	A description of all covariates tested
☐	☒	A description of any assumptions or corrections, such as tests of normality and adjustment for multiple comparisons
☐	☒	A full description of the statistical parameters including central tendency (e.g. means) or other basic estimates (e.g. regression coefficient) AND variation (e.g. standard deviation) or associated estimates of uncertainty (e.g. confidence intervals)
☐	☒	For null hypothesis testing, the test statistic (e.g. *F*, *t*, *r*) with confidence intervals, effect sizes, degrees of freedom and *P* value noted<br>*Give P values as exact values whenever suitable.*
☒	☐	For Bayesian analysis, information on the choice of priors and Markov chain Monte Carlo settings
☐	☒	For hierarchical and complex designs, identification of the appropriate level for tests and full reporting of outcomes
☒	☐	Estimates of effect sizes (e.g. Cohen's *d*, Pearson's *r*), indicating how they were calculated

Our web collection on statistics for biologists contains articles on many of the points above.

Software and code

Policy information about availability of computer code

Data collection	See Methods for details of all equipment used for data collection. Briefly, immunohistochemistry images were acquired on microscopes using Zeiss imaging software (Axiovision 4.8 and ZEN blue). Large scale histological analyses were performed using a Zeiss AxioScan Z1 slide scanner. FRET images were collected in a custom built NIKON microscope. Human MRI/DTI Imaging data was collected using a 3.0T Philips Achieva TX scanner (Philips Medical Systems, Best, The Netherlands). EM was performed using a JEOL 1400 Plus transmission electron microscope.
Data analysis	JMP version 16 and 17.1, and Graphpad Prism v9 were used for most data analyses, statistics, and graph creation. FRET images and analyses were performed using MetaMorph software ver. 7.10.5 (Molecular Devices). MatLab (ver 2011a; Mathworks, Natick, MA, USA) software was used to perform image processing and data analyses. EthoVision XT (version 14) was used to analyzed behavioral data.

For manuscripts utilizing custom algorithms or software that are central to the research but not yet described in published literature, software must be made available to editors and reviewers. We strongly encourage code deposition in a community repository (e.g. GitHub). See the Nature Portfolio guidelines for submitting code & software for further information.

Data

Policy information about availability of data

All manuscripts must include a data availability statement. This statement should provide the following information, where applicable:

- Accession codes, unique identifiers, or web links for publicly available datasets
- A description of any restrictions on data availability
- For clinical datasets or third party data, please ensure that the statement adheres to our policy

All raw data and the datasets generated during and/or analyzed during the current study are available from the corresponding author on reasonable request.

Research involving human participants, their data, or biological material

Policy information about studies with human participants or human data. See also policy information about sex, gender (identity/presentation), and sexual orientation and race, ethnicity and racism.

Reporting on sex and gender	This study reports on the molecular and cellular mechanisms that underlie an ultra-rare disorder. Approximately 30 individuals have been identified throughout the world, and only two males were analyzed by MRI and DTI for this study. Therefore there were no considerations based on sex or gender due to the low number of patients.
Reporting on race, ethnicity, or other socially relevant groupings	Controls based on race, ethnicity, or other socially relevant groupings were not possible due to the low number of patients included in this study.
Population characteristics	See above.
Recruitment	Participants were not recruited. The families of individuals with ANDS reached out to the corresponding author.
Ethics oversight	All procedures were performed under the ethical approval by the Institutional Review Board (IRB) at Albert Einstein College of Medicine and are described in IRB protocol #2011-320.

Note that full information on the approval of the study protocol must also be provided in the manuscript.

Field-specific reporting

Please select the one below that is the best fit for your research. If you are not sure, read the appropriate sections before making your selection.

☒ Life sciences ☐ Behavioural & social sciences ☐ Ecological, evolutionary & environmental sciences

For a reference copy of the document with all sections, see nature.com/documents/nr-reporting-summary-flat.pdf

Life sciences study design

All studies must disclose on these points even when the disclosure is negative.

Sample size	Sample sizes were selected based on previous experience of the types of experiments performed and the manitudes of results expected. N-values were selected based on whether they would be sufficient to obtain results with valid statistical power (typically a=0.05 b=0.85).
Data exclusions	No data were excluded from any dataset.
Replication	All experiments were performed in at least three cohorts separated by time. Behavioral analyses were performed in at least three different litters, cell culture experiments were performed from three different platings and so on. If at least three different cohorts did not present consistent results, then experiments were dropped. Therefore all replications were successful for the experiments presented in this study.
Randomization	Allocation was random
Blinding	All experiments were perform blind to conditions/genotypes/treatment. In all cases, one of the authors was assigned the coded key to unblind the experimenter at the end of data analyses. In some cases, experimenter asked for which coded samples belonged to which experimental group.

Reporting for specific materials, systems and methods

We require information from authors about some types of materials, experimental systems and methods used in many studies. Here, indicate whether each material, system or method listed is relevant to your study. If you are not sure if a list item applies to your research, read the appropriate section before selecting a response.

Materials & experimental systems

n/a	Involved in the study
☐	☒ Antibodies
☒	☐ Eukaryotic cell lines
☒	☐ Palaeontology and archaeology
☐	☒ Animals and other organisms
☒	☐ Clinical data
☒	☐ Dual use research of concern
☒	☐ Plants

Methods

n/a	Involved in the study
☒	☐ ChIP-seq
☒	☐ Flow cytometry
☐	☒ MRI-based neuroimaging

Antibodies

Antibodies used

AIDA-1(1A11), Mouse, in-house; AIDA-1(2B22), Mouse, in-house; AIDA-1(2K9), Chicken, 1µg/mL, in-house, APC(CC-1), Mouse, 1:1000, CatNo:OP80, Millipore Sigma; GAPDH, Rabbit, 1:1000, CatNo:2118, Cell Signaling; MBP, Chicken, 1:1000, CatNo: CPCA-MBP, EnCor; Olig2, Rabbit, 1:1000, CatNo:AB9610, Millipore Sigma; PDGFRα(CD140α), Mouse, 1µg/mL, CatNo:558774, BD Pharmigen; PDGFRα(CD140α), Goat, 1µg/mL, CatNo:AF1062, R&D, PSD95, Mouse, 1:1000, CatNo:75-028, NeuroMab; PV, Rabbit, 1:1000, CatNo: AB15736, Millipore Sigma; Sox10, Mouse, 0.5µg/mL, Developmental Hybridoma Bank; SST, Rabbit, 1:1000, CatNo: HPA019472, Millipore Sigma; Tubulin, Mouse, 1;1000, Thermo Fisher.

Validation

AIDA-1 mouse antibodies 1A11, 2B22, and 2K9 were previously validated in knockout mice (Tindi JO et al 2015) and following shRNA-mediated knockdown of Anks1b in cultured rat primary neurons (Carbonell AU, 2019). Antibody APC(CC1) was validated to bind specifically to Quaking-7 in a previous publication (Bin JM 2016, J Neuroschem). GAPDH, Rabbit by Cell Signaling Tech was validated by size using Western blots and enrichment in mitochondria using immunofluorescence in the product description page. Antibodies to MBP (Chicken, Encorbio), Olig2 (Rabbit, Millipore Sigma), PDGFRa (Mouse, BD Pharmingen and Goat, R&D), PSD95 (Mouse, Neuromab), PV (Rabbit, Millipore Sigma), Sox10 (Mouse, Developmental Hybridoma Bank) and SST (Rabbit, Millipore Sigma) were validated by Western blot (for size) and Immunofluorescence (by expected location) on the production description page.

Animals and other research organisms

Policy information about studies involving animals; ARRIVE guidelines recommended for reporting animal research, and Sex and Gender in Research

Laboratory animals

Both mice and rats were used in this study. All transgenic mice were on a C57Bl6J background. All behavioral testing was performed on mice aged between 3-4 months, except in the Clemastine rescue assays, where behavioral tests were peformed in 7-month old mice. Experiments throughout the study were performed in different aged mice as indicated in each experiments. Both pregnant mice and pregnant Sprague-Dawley rats were used to isolate primary cell tissue for culturing, typically from E18-E21 embryos or P1-3 postnatal pups.

Wild animals

No wild animals were used.

Reporting on sex

Mice of both sexes were evaluated in behavioral studies. Sex-based analyses were performed for all behavioral studies reported. However, no statistical differences were observed between males or females in any of the mouse behavioral studies where analysis by sex was possible, so only lumped data is shown. For rat studies, only female rats were used because we needed embryonic tissue or newborn pups from pregnant females to create primary cell cultures.

Field-collected samples

no field-collected samples were used in this study.

Ethics oversight

All protocols were approved by Albert Einstein College of Medicine's Institutional Animal Care and Use Committee (IACUC) in accordance with National Institutes of Health guidelines.

Note that full information on the approval of the study protocol must also be provided in the manuscript.

Magnetic resonance imaging

Experimental design

Design type

Resting-state - Please refer to materials and methods for more in-depth description of tests.

Design specifications

Data was collected in one block ~30-40 min of length.

Behavioral performance measures

Resting-state tests. No activities were requested.

Acquisition

Imaging type(s): Diffusion Tensor Imaging-

Field strength: 3 Tesla

Sequence & imaging parameters: Imaging data was collected using a 3.0T Philips Achieva TX scanner (Philips Medical Systems, Best, The Netherlands) with a 32-channel head coil. Structural 3D T1-weighted (T1W) magnetization-prepared rapid acquisition of gradient echo imaging was performed with TR/TE/TI = 8.5/3.9/900 ms, α =8°, SENSE factor 2.0/2.6 in SI/RL directions, 1 mm3 isotropic resolution, 240 × 240 × 220 matrix. Diffusion tensor imaging (DTI) data was acquired using 2D single-shot spin echo EPI with 32 diffusion-weighting directions at b=800 s/mm2 and TE = 56ms, TR = 7.6s, SENSE factor=2.9, 2 mm3 isotropic resolution, 128 × 128 matrix, 70 slices. An auxiliary field map scan was acquired using FOV=256mm, 4.0 mm3 isotropic resolution, TR=26ms, TE/DTE= 2.5/2.3ms, α = 26o. The field map is used to correct susceptibility-induced distortions in echo-planar (diffusion) scan. (FROM METHODS)

Area of acquisition: Whole brain scan

Diffusion MRI: ☒ Used ☐ Not used

Parameters: Please refer to materials and methods for more in-depth description of tests.

Preprocessing

Preprocessing software: Please refer to materials and methods for more in-depth description of tests.

Normalization: Please refer to materials and methods for more in-depth description of tests.

Normalization template: Please refer to materials and methods for more in-depth description of tests.

Noise and artifact removal: Please refer to materials and methods for more in-depth description of tests.

Volume censoring: *Define your software and/or method and criteria for volume censoring, and state the extent of such censoring.*

Statistical modeling & inference

Model type and settings: Please refer to materials and methods for more in-depth description of tests.

Effect(s) tested: Please refer to materials and methods for more in-depth description of tests.

Specify type of analysis: ☐ Whole brain ☐ ROI-based ☐ Both

Statistic type for inference (See Eklund et al. 2016): Please refer to materials and methods for more in-depth description of tests.

Correction: Please refer to materials and methods for more in-depth description of tests.

Models & analysis

n/a	Involved in the study
☒	☐ Functional and/or effective connectivity
☒	☐ Graph analysis
☒	☐ Multivariate modeling or predictive analysis
